# Road Traffic Injury Trends in the City of Valledupar, Colombia. A Time Series Study from 2008 to 2012

**DOI:** 10.1371/journal.pone.0144002

**Published:** 2015-12-10

**Authors:** Jorge Martín Rodríguez, Rolando Enrique Peñaloza, José Moreno Montoya

**Affiliations:** 1 Cendex. Pontifícia Universidad Javeriana. Bogotá, Colombia; 2 Universidad del Rosario School of Medicine and Health Sciences. Bogotá, Colombia; Indian Institute of Toxicology Research, INDIA

## Abstract

**Objective:**

To analyze the behavior temporal of road-traffic injuries (RTI) in Valledupar, Colombia from January 2008 to December 2012.

**Methodology:**

An observational study was conducted based on records from the Colombian National Legal Medicine and Forensic Sciences Institute regional office in Valledupar. Different variables were analyzed, such as the injured person’s sex, age, education level, and type of road user; the timeframe, place and circumstances of crashes and the vehicles associated with the occurrence. Furthermore, a time series analysis was conducted using an auto-regressive integrated moving average.

**Results:**

There were 105 events per month on an average, 64.9% of RTI involved men; 82.3% of the persons injured were from 18 to 59 years of age; the average age was 35.4 years of age; the road users most involved in RTI were motorcyclists (69%), followed by pedestrians (12%). 70% had up to upper-secondary education. Sunday was the day with the most RTI occurrences; 93% of the RTI occurred in the urban area. The time series showed a seasonal pattern and a significant trend effect. The modeling process verified the existence of both memory and extrinsic variables related.

**Conclusions:**

An RTI occurrence pattern was identified, which showed an upward trend during the period analyzed. Motorcyclists were the main road users involved in RTI, which suggests the need to design and implement specific measures for that type of road user, from regulations for graduated licensing for young drivers to monitoring road user behavior for the promotion of road safety.

## Introduction

In recent years road-traffic injuries (RTI) have constituted a public health problem that has become one of the major topics on the agenda of the World Health Organization (WHO). The magnitude of the problem was evidenced according to figures of increased mortality rates, quality-adjusted life years, lost life years, and disability. [1,2] For 2013 WHO reports estimate around 1.24 million deaths occurring annually, [3] and it projects that, if no efforts are made, the problem will be the fifth cause of disability and comorbidity, only surpassed by chronic conditions. [2]

In addition, RTI generate social, family, and emotional impacts, including bereavement, orphanhood and high healthcare costs. [4,5] Medium-income and low-income countries are the most affected, with mortality rates from 18.3 to 20.1 per 100,000 inhabitants, respectively. Furthermore, medium-income and low-income countries contribute 49.6% of the total number of deaths but they just own 9.2% and 38.7% of the vehicle fleet correspondingly. [2,6,7,8,9]

Structurally, the phenomenon appears to be associated with individual factors such as the driver’s alcohol consumption and his or her non-use of safety features, for example, seat belts in cars or helmets for motorcyclists. Contextual variables have also been described, mainly, violation of speed limits, problems associated with vertical and horizontal signaling, roads in poor condition (including poor performance when the pavement is wet), an increased automobile fleet and lack of visibility.[2] Likewise, efficient vehicle characteristics have been reported, mainly, poor mechanical conditions. As to regulation, the evidence points to deficient or nonexistent traffic rules and regulations or even to the regulating authority’s lax and permissive attitudes. Cultural norms and behaviors such as “*mototaxismo*” (“motorcycle cab service”) were also associated. [1–3,10,11]

At a local level, in Colombia with 5,792 deaths during 2011 (an average rate of 12.6 per 100,000 inhabitants) the RTI were the second highest cause of death among all external causes. Inside the country the highest rates varied from 22.2 to 32. For the state of Cesar, the twelfth-most populated Colombian state, the figure reached 239 deaths, out of which 63 cases occurred in its largest city and the state capital: Valledupar. As concerns non-fatal road injuries, Colombia recorded 40,806 road incidents, out of which 946 were reported in Cesar, 502 having occurred in Valledupar. Among the main circumstances surrounding road traffic fatalities are the disobeying of traffic rules and regulations (42%), exceeding the speed limit (32%), and the presence of mechanical failures (8%). For non-fatal injuries, the main causes are disobeying traffic rules (64%), exceeding the speed limit (21%), and drunken driving (7%). The actions that the government has proposed for reducing the magnitude of this problem in Colombia in recent years have not had the desired effect. [12]

Taking into account the above, RTI represent an important public problem in Colombia, especially aggravated by the low effectiveness of regulations and increasing motorcycle and motor-vehicle sales. Due to the above, this paper aims to analyze road-traffic injury behavior and its trend in the city of Valledupar from 2008 to 2012.

## Material and Methods

This study was conducted in the city of Valledupar, the state capital of Cesar, Colombia. The city is located in the northeastern region and it is an important agricultural, cattle-raising and agro-industrial center for the country. Its economy is based on agricultural production, food processing, and livestock and it is one of the major music, cultural, and folk centers of the country. [13] In 2012, Valledupar had around 423,000 inhabitants, 83% of whom lived in the urban area. The gender distribution indicates that 51.3% of the population is male. [14]

### Data source and statistical analysis

The information used in this study is based on the medical records of the Colombian National Legal Medicine Information System (CNLMIS). The database for unintentional violent events was analyzed, more specifically for RTI. Deaths due to road traffic crashes as well as other injuries were excluded. [15] Initially the data was described using means and frequencies and tabulated according to variables such as sex, age, and education level and type of road user. Further analyzed were the time frame (day, month, and year of the occurrence); the place (the particular scene of the incident and the area) and the circumstances of the crash and the vehicles, the kind of event, and possible errors associated with the occurrence.

The time series analysis was made by building autoregressive integrated moving average (ARIMA) models according to the methodology proposed by Box-Jenkins. [16] In this additive model, the mean of the RTI is adjusted for each time period by weighting the influence of past observations and a linear combination of random errors with mean zero and constant variance. Constant variance represents the disturbances observed but not directly related to the process per se but rather to unobserved variables.

In practice, these models have six parameters (p, d, q) X (P, D, Q), where p represents the number of past observations that significantly affects the current mean; d represents the polynomial degree of the trend in the time pattern of the RTI; and q represents the number of disturbances as mentioned above. The capital letters indicate the same but for long time periods where the seasonal effect would be seen. For this analysis such effect was considered as annual. The model was evaluated through goodness of fit and fitness testing. All the analyses were carried out using the STATA 12 statistical package. The variables sex and age were solely used for descriptive purposes.

This research is considered safe according to Colombian Ministry of Health Decree 8430 (1993). It was approved by the Universidad Javeriana University (Bogotá campus) Ethics Committee and by its Center for Development Projects. All the information sources were secondary; the researchers did not have access to identification data.

## Results

**Fig 1:**
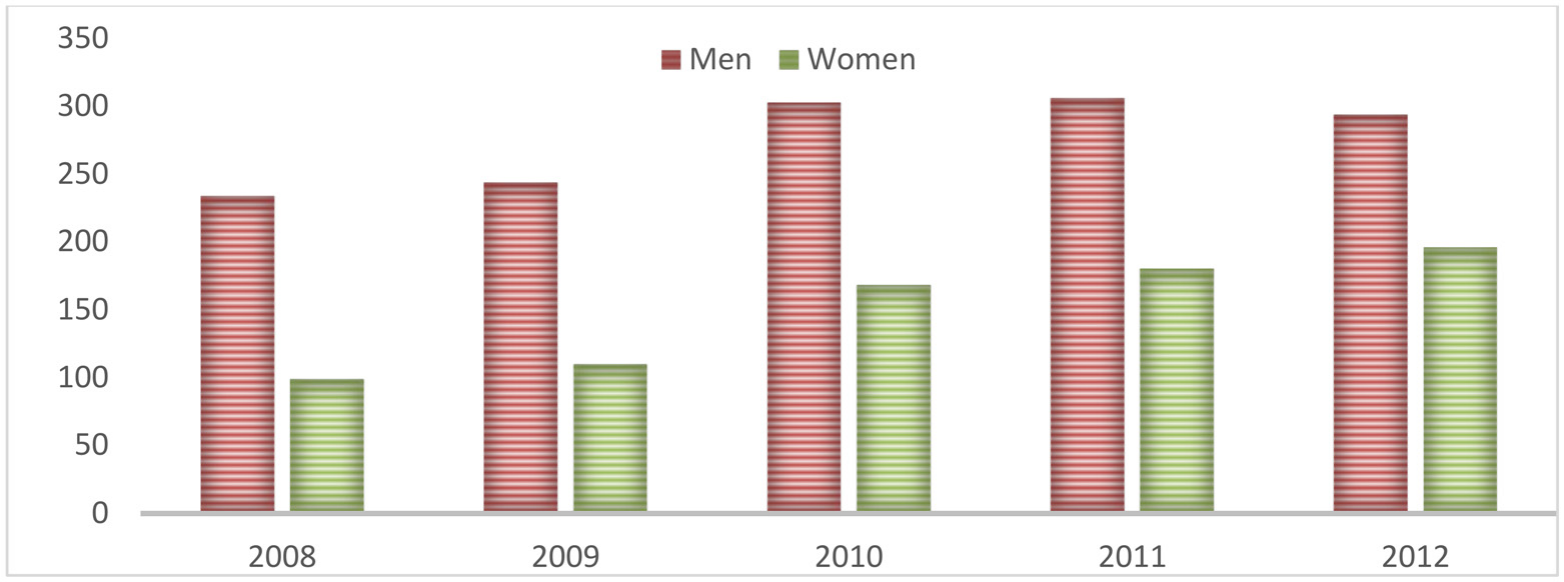
Monthly distribution of traffic road injuries in Valledupar, by sex from 2008 to 2012.

Records were analyzed for a total of 60 months from January 2008 to December 2012, showing an average of 104.93 road incidents per month (29.89 SD) varying from 43.8 to 168.4 (84.4–127.6, IR; median: 104.98). 64.9% of the total events involved men with an approximate ratio of 2 men to 1 woman (see Fig 1). The age parameter showed that 21.7% of the injured were under 18 years of age, 82.3% from 18 to 59 years of age, and 6.9% over 60 years of age; the average age was 35.4 years of age; being lowest among motorcyclists (32.3) who were the road users most involved in RTI (69%), followed by pedestrians (12%). The educational level parameter showed that 20.7% had only had a primary education or less and that 51.5% had a secondary education level.

RTI were most frequent in the first month of the year in 2008, 2010, and 2011. Sunday (18.6%) appeared to be the most violent day, followed by Saturday (15.6%), Monday (15.2%), and Friday (14.1%). 93% of the total events occurred in the urban area. 92% of the events occurred on public roads. According to CNLMIS records, possible circumstances of errors leading to the occurrence of the RTI were: violation of traffic rules (70.5%), exceeding the speed limit (10.5%), drunken driving (2.5%), possible mechanical failures (0.9%), poor road conditions (0.7%), running a traffic light (0.6%), driving the wrong way on a one-way street (0.6%), and others (13.7%). (Data not shown).

In general terms, the RTI frequency seems to have no trend. Annually, the average occurrence of road-traffic crashes is similar (p-value: 0.224) (see Table 1);

**Table 1:** Road-traffic occurrence per year.

Year	Total	Average	Minimum	Maximum
2008	1041.4	86.8	43.8	156.4
2009	1079.5	90.0	45.7	134.2
2010	1401.1	116.8	83.3	151.7
2011	1411.0	117.6	75.5	168.4
2012	1386.4	115.5	90.7	144.6

**Fig 2:**
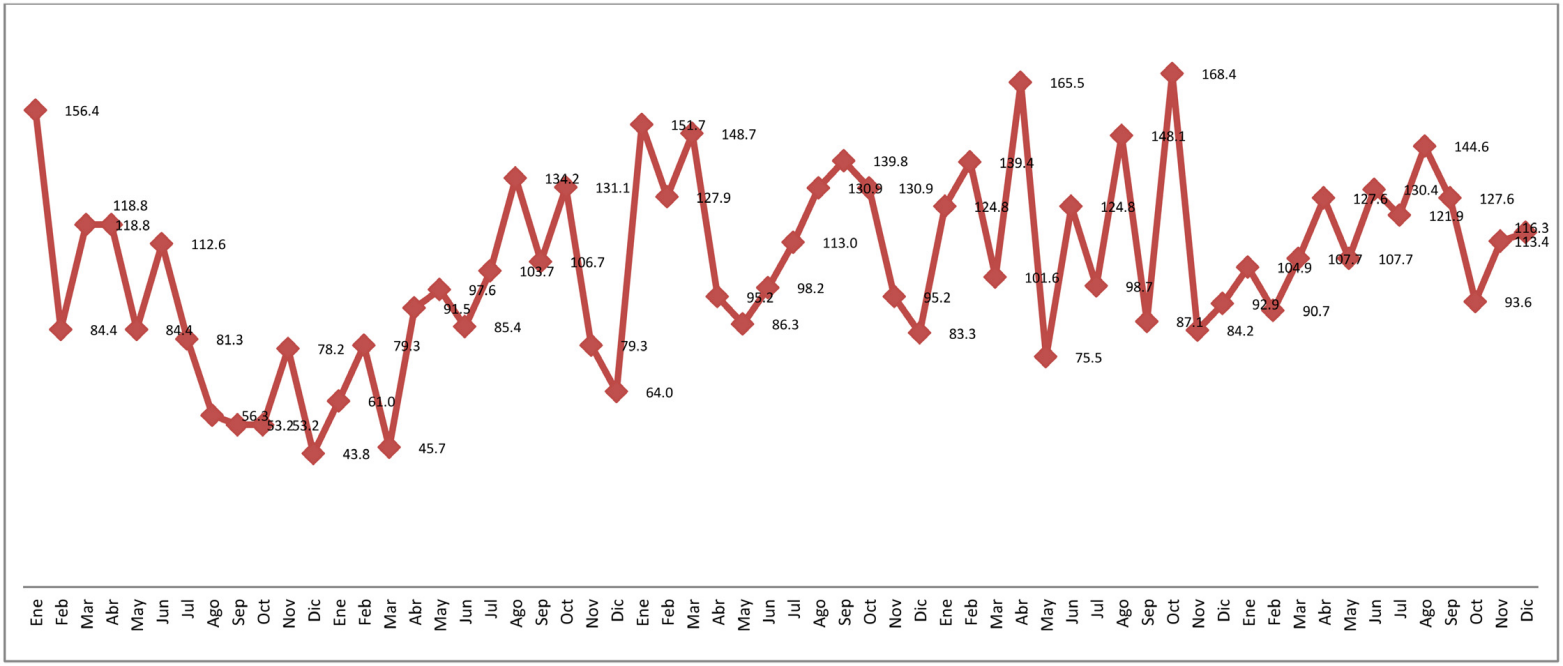
Road-traffic crashes by month.

However, this result may be related to the fact that the time series has an important decrease followed by a significant increase around the 19th month, which can be easily appreciated in the graphic representation (see Fig 2).

**Fig 3:**
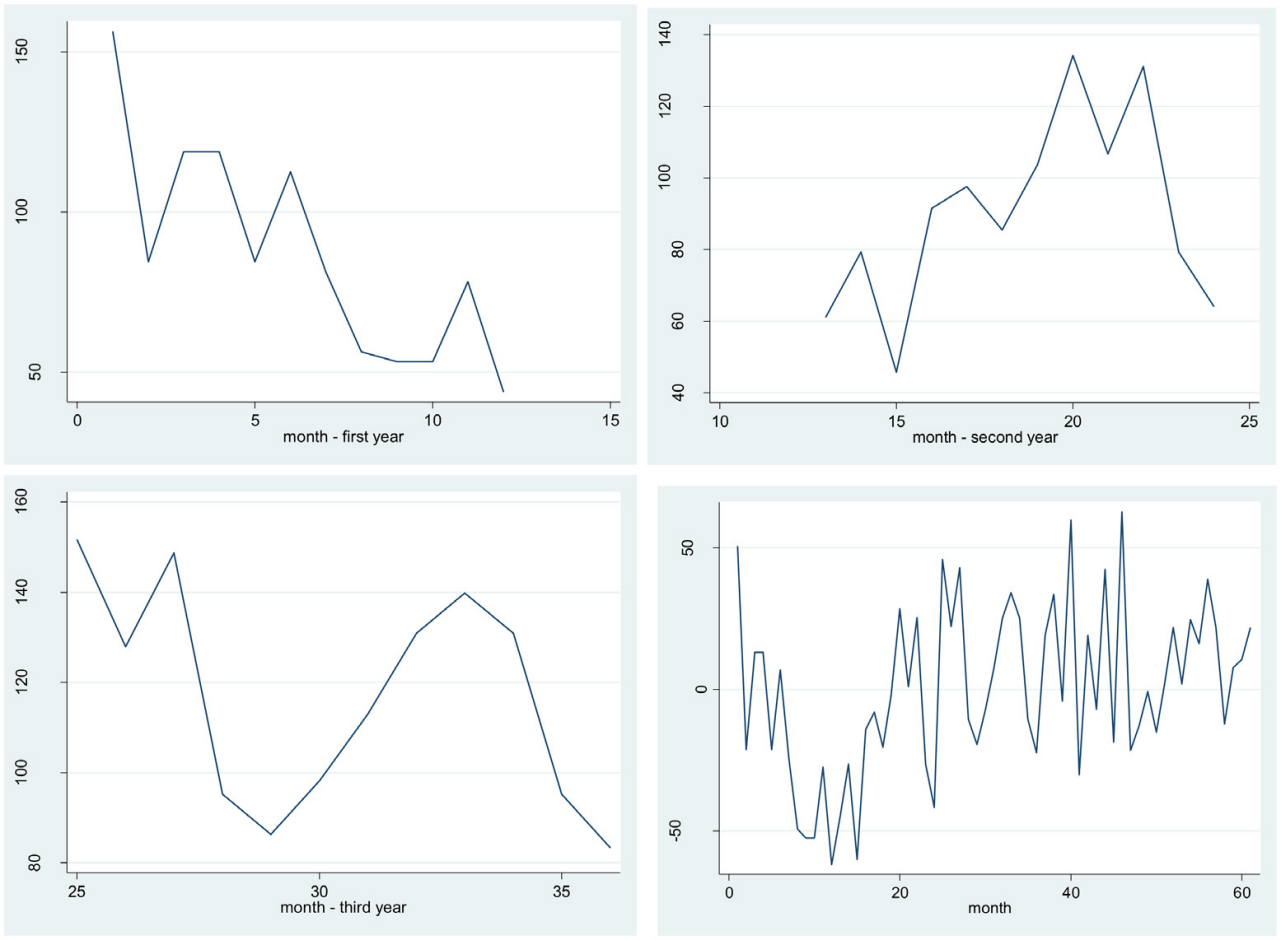
Monthly variation of the series for the first three years and differentiated time series.

A seasonal pattern can also be verified. The visual examination of the time series enables recognizing some peaks with a similar apparent frequency. In fact, this behavior corresponds to an annual increase / decrease of the phenomenon, which has occurred, on an average, during the same time periods, in general, around month eleven. The time series was differentiated to confirm the presence of a trend and a seasonal pattern, by eliminating the annual data. A graphical representation of the seasonal pattern and of the differentiated series may be appreciated in Fig 3.

Analytically speaking, the existence of the seasonal and trend effects was also verified by using the Dickwy-Fuller test [17] (P-value = 0.999). Taking the above into account, an ARIMA model was proposed, aimed at explaining the variation structure of the time series. This process yielded a seasonal model that verifies two important characteristics of the monthly number of recorded road-traffic crashes in Valledupar. First, the variation in each month depends on the previous figure up to five months before. Second, there is an important seasonal component that indicates that the previous year’s figure significantly affects the next year’s average (memory process). Important random disturbances were also identified, involving at least the two months prior to the month being analyzed (see Table 2). The model was significantly different from the empty model (p < 0.001).

**Table 2:** Adjusted ARIMA model ^δ^.

Regressive Parameters, by month^€^	Coefficient	95%	CI	p
1	-0.41	-0.91	0.11	0.124*
2	-0.33	-0.85	0.18	0.201*
3	-0.56	-0.88	-0.24	0.001
4	-0.55	-0.93	-0.15	0.007
5	-0.46	-0.88	-0.04	0.033
Random disturbances, by month^¥^				
1	-1.28	-1.89	-0.67	0.000
2	0.61	0.02	1.21	0.042
Seasonal regressive parameters, by month^β^				
12	-0.64	-1.00	-0.28	0.001

^δ^ The parameters correspond to the observed effects for a differentiated series, i.e., a series where the trend and seasonal deterministic patterns were removed first, in order to improve the stochastic estimation in the stochastic model.

* Non-significant at a level of 0.05

^€^ Previous months identified as important for predicting the variation of the current month

^¥^ Disturbances occurred during a previous month, which affect the current realization of the phenomenon

^β^ Seasonal effect (past year value) that affects the current value of the time series

Using the above notations, the final model may be written as an ARIMA model (5,1,2) X (1,1,0,12). This model shows that the time variation pattern observed in the series of road crashes in Valledupar is not merely random. The significant coefficients in the model are related to the effect that previous observations (values of the series) have on future values, i.e., their influence. This may be statistically understood as a self-correlation; however, taking the time variable into account, it may also read as a solid tool for predicting future values based on previous observations. The disturbances, on the other hand, point to effects not derived from the series itself. The year parameter indicates that the annual behavior is an important factor to be analyzed in road-traffic crashes in the city.

## Discussion

This study was based on the local records for road crashes in the city of Valledupar, Colombia. An ARIMA model was used to describe the structure of variations in time of the rate of road-traffic crashes. A significant influence of the previous months’ figures on the current month’s figures was found, more specifically, the third, fourth and fifth months before the month analyzed. Two significant parameters were also associated with random disturbances for the first and second months before the current month. A seasonal (yearly) influence was further verified.

A possible explanation for the observed time variation pattern may be derived from the existence of two circumstances. One is the process memory, i.e., the effect of the previous number of crashes on the near future; the second one is the existence of unobserved variables. In fact, the disturbances that appear with significant effects on the adjusted model solely as random variations may be associated with the impact of external influences and most importantly during the last two months where the process memory appears to be non-significant. Both circumstances are interesting from the perspective of public health. Firstly, acknowledging a time pattern may represent a corner stone for the surveillance and intervention of this public problem. This, in fact, may establish the principles of a moderate or time-regulated intervention plan. That consideration has been made in many other areas of health research and surveillance, and in many contexts [18,19,20]. Secondly, it shows the need for more in-depth RTI research.

RTI are a group of multifactorial origin events; they are a product of the combination of factors that interact and are interconnected so as to make them appear. It is also important to recognize that these factors are the product of distal causes or determinants. Below is a brief discussion of the possible association of the findings with human determinants (behaviors, attitudes and practices of lack of respect for traffic rules), social determinants (legislation implementation and enforcement problems, insufficient force for regulations supervision and monitoring, lax-term motorcycle sales), vehicle determinants (motorcycles are the vehicles most affected in the different road incidents in the city), and environmental determinants (lack of vertical and horizontal signaling, designs and characteristics of roads, etc.). These are the determinants mainly related to the occurrence of RIT in Valledupar.

About 80% of such injuries are suffered by vulnerable road users (motorcyclists and pedestrians) and most (45%) occurred on weekends. The motorcyclists were the main road users involved in RTI. The rapid increase in the number of vehicles in Colombia, especially motorcycles [11–13,16] may have a relation to recklessness; and the lack of experience and risky behavior of many motorcyclists are probably related to the boost in this public health problem[21,22]. The average age of those affected is lower than the average age of other road users, which is concordant with other reported results for these users. [23] In Colombia, before 2013, there were no clear regulations for punishing drunken drivers; it is likely that the observed increase in injuries on weekends was associated with such circumstances; additionally, there are usually fewer control measures on holidays when the cultural aspects of the community also play an important role. [24]The above human, social and vehicle determinants generated in RTI are in part, consistent with Haddon’s vision. [25,26]

To improve the understanding of human determinants, qualitative techniques were implemented in this research (focus groups, in-depth interviews, and observations) with the support and advice of an anthropologist. The authors consulted health authorities, as well as transit, government, education, and public works officials professionals involved in pre-hospital healthcare services, auto parts sales persons, individuals who had suffered road traffic injuries in the past, among others, who all consistently reported that the increase in the volume of RTI in the first quarter of the year is a result of the absence of prevention and control measures at that time of the year. It is only in April that bureaucratic mechanisms leading to the restoration of control measures are started up, through which the mayor engages a specialized group of traffic police to conduct prevention, education and control measure reinforcement processes in the city. [27,28]

Another key element for reducing RTI among the inhabitants of Valledupar is the study and improvement of the road network. WHO has acknowledged that this is an essential factor in the generation of RTI, from slight to fatal injuries. [2] In a preliminary observation, the findings of Patiño et al. identified that in Valledupar there are great limitations in vertical and horizontal signaling, and in the maintenance of the urban road network. [29] As is seen in other studies, the road auditing implementation process in Valledupar has been effective in lowering RTI sites. [30,31,32] It establishes characterizations of the road network conditions, for the purpose of taking corrective actions against identified risks in the city. The environmental determinants potentially associated with RTI are clear here.

This study has some limitations. The series used is very short for a precise estimation of effects. That may compromise the accuracy of the estimated parameters, [33] but not their validity. Another constraint is the origin of the information; secondary data was used for this study, so the quality and integrity of the information depends on how it was originally recorded. The retrospective and ecological perspectives of this analysis also represented some limitations; therefore, no causality inference can be made from the findings. Along the same lines, the predictions are quite limited as they were not the main goal of the proposed methodology. Another important issue to take into account is the fact that the information used was restricted to the figures themselves.

Nevertheless, this effort has resulted in one of the first papers describing a seasonal pattern for road-traffic injuries in a developing country such as Colombia. The results may, therefore, contribute useful information on the behavior of the mortality and morbidity derived from RTI in similar regions. Also, the results presented here may represent a useful tool for planning and developing local intervention actions. In the same manner as the model was used to identify the variation in the phenomenon for time, the methodology used may help assess changes in the figures, their trend or seasonal behavior due to regulatory changes or due to any other kind of intervention.

As a public action to help find a solution to this problem, a workshop was conducted with stakeholders. The workshop presented three goals and eight strategic objectives.

The three goals were: a) to achieve the highest level of road safety throughout the city of Valledupar: by engaging the citizens of Valledupar to play their part as those mainly responsible for their own safety and that of others on the city streets; b) to attain an integrated approach to road safety cooperation involving local government and the Ministry of Health, the Ministry of Traffic, the Ministry of Government, the Ministry of Education, the Colombian National Police, other government sectors, and the private sector; c) to understand that this is a shared responsibility: at national, state, and municipal levels.

The eight strategic objectives were:

1. To improve education and training for road users—It is important to enhance the quality of the training system and of the licensing process, with particular emphasis on young drivers and motorcyclists. The city should work on developing one sole educational and training road safety strategy. Emphasis should be made on compliance with the use of protective elements (helmets, seat belts).
2. To have better prevention and control measures, to be pursued in the medium and long term—_This action should involve broad social participation, including the road users. [10,11,13]
3. To achieve more compliance with traffic rules and regulations—The city should develop campaigns and increase installed capacity, to improve compliance capacity. It should also implement and enforce the legislation; for example, graduated licensing regulations for young drivers, as are seen in several countries around the world [1,34,35,36] Legal implementation is as challenging as it is important because it suggests previous evidence. [2]
4. To augment resource allocation for road safety—Local and regional government agencies should allocate a larger portion of the budget for developing the city road safety plan; such resources would also be aimed at designing and implementing environmental improvements, as well as road and street infrastructures in the city.
5. To improve emergency services and care to persons suffering RTI. The city should propose a global action strategy for persons injured in traffic accidents, including first aid;
6. To mainly focus on the most vulnerable road users—The city will work to improve the safety of motorcyclists and pedestrians, emphasizing proper behavior, using protection elements, vehicle safety, and ensuring an infrastructure for pedestrians;
7. To better the planning process for developing the city road network—The city will work to develop a long-term plan that meets road infrastructure safety requirements;
8. To establish the Road Safety Observatory—The city will work to create a road safety observatory in order to track the behavior of RTI as to the dead and injured in traffic accidents there. This Observatory would improve the injury surveillance system, which would help to detect patterns and trends, in order to orient specific interventions;
